# Network Pharmacology Unveils Multi-Systemic Intervention of *Panax notoginseng* in Osteoporosis *via* Key Genes and Signaling Pathways

**DOI:** 10.2174/0118715303335018241107084224

**Published:** 2025-01-09

**Authors:** Qiyue Wang, Xiaoping Wang, Kezhou Wu, Weiwei Wu, Zhantu Wei, Weili Feng

**Affiliations:** 1 Sports Medicine Center, Department of Orthopaedic Surgery, The First Affiliated Hospital of Shantou University Medical College, Shantou, Guangdong Province, People’s Republic of China;; 2 Department of Orthopaedics, Xiaolan People’s Hospital of Zhongshan, Zhongshan, Guangdong Province, People’s Republic of China

**Keywords:** Network pharmacology, *Panax notoginseng*, osteoporosis, fracture, Ginseng, gene expression

## Abstract

**Background:**

*Panax notoginseng* (Burk.) F. H. Chen (PN) is a traditional Chinese medicine that has been applied to prevent and treat osteoporosis. The mechanism of PN for osteoporosis remained a mystery.

**Objective:**

The objective of this study was to reveal the therapeutic effect and illuminate the possible mechanism of PN for osteoporosis.

**Methods:**

The Traditional Chinese Medicine Database and Analysis Platform was searched to screen the potent ingredients of the PN and to analyze the potential therapeutic targets for osteoporosis. We excavated four disease databases to collect osteoporosis-related genes. After integrating the gene expression profile of osteoporosis and the chemical-protein data of PN, a protein-protein interaction network was constructed to demonstrate the interactions among the gene products. GO function, KEGG pathway, and docking analyses were executed.

**Results:**

Network pharmacology obtained 31 active ingredients and 134 targets for the treatment of osteoporosis. The key components were beta-elemene, quercetin, methyl palmitate, oleic acid, and hexanal. The results of GO and KEGG analyses showed that *Panax notoginseng* was beneficial for osteoporosis by influencing the main pathways including AGE-RAGE signaling pathway in diabetic complications, TNF signaling pathway, IL-17 signaling pathway, p53 signaling pathway, NF-kappa B signaling pathway, PI3K-Akt signaling pathway, MAPK signaling pathway, FoxO signaling pathway, and Wnt signaling pathway, modulating inflammation, metabolism, cell proliferation, cell survival, growth and angiogenesis. *Panax notoginseng* intervened in osteoporosis through multi-components, multi-targets, and multi-pathways.

**Conclusion:**

This study illustrates the mechanism of *Panax notoginseng* for osteoporosis, providing broader insights for novel research and developments of the Panax species for osteoporosis.

## 1 INTRODUCTION

Osteoporosis is a systemic skeletal condition characterized by reduced bone mass and microarchitectural deterioration, which increases bone fragility and the risk of fractures [1]. Traditionally, it is recognized by a bone mineral density (BMD) T score of ≤ -2.5 [2]. Predominantly affecting adults, especially postmenopausal women, osteoporosis is often asymptomatic in its early stages. Despite the lack of early symptoms, the condition can lead to acute fractures after a fall, which in turn can result in lifelong disability, increased mortality rates, and a significant reduction in the quality of life for patients [3].

The physiological balance of bone homeostasis and the pathological process of osteoporosis involve interlaced signaling pathways and numerous regulatory targets. The paradigm of osteoporosis-related signaling pathways that are being widely studied included the Wnt signaling pathway, OPG/RANK/RANKL signaling pathway, MAPK signaling pathway, PI3K/Akt signaling pathway, *etc.* [4, 5]. Based on these modulatory mechanisms of bone resorption and bone matrix formation, a series of therapeutic agents targeting osteoporosis have been designed and emerged for clinical application, classified as antiresorptive and anabolic drugs. The antiresorptive drugs, including bisphosphonates, estrogen, selective estrogen receptor modulator (SERM), calcitonin, cathepsin K inhibitors, denosumab, and romosozumab, inhibit the recruitment and activation of osteoclasts, diminishing the bone remodeling rate and partially reversing the wedge-shaped deficit in bone micro-architecture. Represented by teriparatide, abaloparatide, and romosozumab, bone-forming or anabolic drugs reinforce bone formations by stimulating periosteum, endosteum, and cancellous bone formation. It is of note that, however, the impact factors and development process leading to osteoporosis are multifold. Any factors that affect bone remodeling and the activity of osteoclasts, osteoblasts and osteocytes can affect the development of osteoporosis. Antiresorptive Drugs, such as Bisphosphonates, can inhibit the activity of osteoclasts through the coupling, while it will also inhibit the activity of osteoblasts and, in turn, slow down bone turnover rate. Anabolic Drugs such as romosozumab not only have the ability to enhance bone formation by upregulating the Wnt pathway but also hold the potency to reduce bone resorption but have a potential tumorigenicity effect [6]. As mentioned above, concerning the complexity of osteoporosis physiopathology, the mainstream single-targeted medicine is far from satisfactory. In the quest for comprehensive therapies for osteoporosis, traditional Chinese Medicine is taking center stage, with related research soaring.

Traditional Chinese medicine is the precious wealth of thousands of years of clinical experience in China, which is characterized by a holistic view and syndrome differentiation. The combination of traditional Chinese medicine and network pharmacology can systematically study the potential targets and pharmacological effects of the drug components in multi-genes, multi-targets, and multi-pathways. *Panax notoginseng* (Burk.) F.H. Chen (Araliaceae), *Panax notoginseng* (PN) for short, documented in “Compendium of Materia Medica” more than 400 years ago, is one of the well-known traditional Chinese herbs [7]. *Panax notoginseng* saponins (PNS) are a group of components obtained from *Panax notoginseng*. Treated with PNS, the bone mass density and microarchitecture in rabbits were remarkedly improved compared with those of the control group [8], indicating that treatment with PNS effectively relieves osteoporosis. PN-derived Yunnan Baiyao (YNBY), a treasure of proprietary Chinese medicine, has demonstrated anti-inflammatory effects in such bone-related diseases as rheumatoid arthritis and osteoporosis [9]. Clinical studies also shown that PNS was able to increase bone density and osteoblast activity.

This study integrated information such as gene expression profile and chemical-protein data of PN through network pharmacological methods to excavate the therapeutic effect of PN for osteoporosis in a comprehensive mode, aiming to reveal the therapeutic effect and illuminate the possible mechanism of PN for osteoporosis.

## 2 MATERIALS AND METHODS

### 2.1 Assessment of ADME Properties and Collection of PN Targets

Traditional Chinese Medicine Systems Pharmacology Database and Analysis Platform, TCMSP in short (https://tcmspw.com/tcmsp.php), is an online platform that calculates and analyses the absorption, distribution, metabolism, and excretion-related properties of natural compounds, which are collectively referred to as ADME properties. The platform not only presents ingredient profiles but also captures the relationship between herbs, targets, and diseases. We searched the TCMSP Database for compounds derived from *Panax notoginseng* without restriction in oral bioavailability (OB) or drug-likeness (DL). After summarizing the constituent of *Panax notoginseng*, we used the same data platform to collect the target genes of PN components. At this stage, we gained each componential molecular ID with its corresponding molecular name, which was matched with the target name (for the retrieved results, please check the *Supplementary Materials*).

### 2.2 Identification of Mutual Genes Between Osteoporosis and PN

We searched databases of GeneCards (https://www.genecards.org/), PharmGkb (https://www.pharmgkb.org/), OM IM (Online Mendelian Inheritance in Man, https://omim.org/), and TTD (Therapeutic Target Database, http://db.idrblab.net/ttd/) for genes related to the search term “osteoporosis.” The search was set to filter out genes not related to Homo sapiens. The disease genes were collected by combining the results of the four databases. Mutual genes between osteoporosis and *Panax notoginseng* were subsequently obtained by taking the intersection of osteoporosis-related genes and PN-associated targets.

### 2.3 Construction of Interaction Networks and Screening of Hub Genes

Cytoscape 3.9.1 software was used to build an “ingredient-target” network. The active ingredients and corresponding targets were represented as “nodes,” and the interactions between nodes were described as “edges.” The potential anti-osteoporosis targets of PN components were imported into the String database. Protein-protein interaction (PPI) networks were established by screening with confidence scores ≥ 0.90, and the resulting PPI network data were analyzed using the CytoNCA plug-in of Cytoscape software. The screening was performed by calculating degree centrality-related indexes, first screening nodes with degree centrality values greater than the median, and then continuing to screen nodes with values greater than the median corresponding to each index based on the initial screening results. Finally, the key targets (hub genes) of PN for osteoporosis were retained.

### 2.4 GO Function Study and KEGG Pathway Analysis

GO, and KEGG enrichment were performed using the DAVID database (https://david.ncifcrf.gov/) to elucidate the gene functions of communal targets between PN and osteoporosis. GO analysis was used to describe the functions of the genes, including cellular components, molecular functions, and biological processes. Both GO and KEGG analyses were statistically significant at *p* < 0.05.

### 2.5 Topological Analyses

The two-dimensional structures of the PN ingredients were downloaded from PubChem (https://pubchem.ncbi. nlm.nih.gov/) and were transformed into three-dimensional structures by ChemOffice software. PyMol software modified the structures by removing ligands, adding hydrogen, and removing water. The online version of CB-Dock (http://clab.labshare.cn/cb-dock/php/index.php) was manipulated to demonstrate the PN ingredients genes products combined with osteoporosis-related genes proteins.

The network pharmacology process is summarized in the graphical abstract.

### 2.6 Extensive Bibliographic Investigation

An extensive literature search was conducted on the five key active substances identified, focusing on experimental studies related to osteoporosis. The search included databases such as PubMed, Web of Science, and Embase, with a publication date range from 2010 to 2024. In the computer search, the following search terms or keywords were applied: beta-elemene, quercetin, methyl palmitate, palmitate, oleic acid, and hexanal. Additionally, each search term was combined with osteoblasts, osteoclasts, osteocytes, bone metabolism, osteoporosis, antioxidant, and anti-inflammatory. Irrelevant results were filtered out, and valuable studies were retained and summarized individually in tables for reference.

## 3 RESULTS

### 3.1 Potential PN Targets for Osteoporosis

TCMSP yielded 194 active targets of *Panax notoginseng*. The osteoporosis-related genes retrieved from GeneCards, TTD, PharmGkb, and OMIM databases were combined to obtain 4,663 disease target genes Fig. (**1a**). By intersecting the PN targets with the disease targets, 134 intersecting targets were obtained Fig. (**1b**), which are the potential targets of Panax ginseng for osteoporosis.

### 3.2 Ingredient-Target Interaction Network

The relationships of PN active ingredients and intersection targets were imported into the Cytoscape software to map the ingredient-target interaction network Fig. (**2**). The pie chart on the left half represents each PN ingredient. The blue squares on the right half denote the intersection targets of disease and PN ingredients. The edges indicate the interaction between them. The connectivity value for an ingredient, defined as the total interactions it has with other network components, is quantified by the number of links it forms with the targets. According to the ranking of the connectivity values, the top five active ingredients are beta-elemene, quercetin, methyl palmitate, oleic acid, and hexanal. They are more closely connected to the intersection targets, which may be the key active ingredients of PN for osteoporosis.

### 3.3 PPI Network and Hub Genes

Potential targets of PN for osteoporosis were uploaded into the STRING data platform with a confidence score of ≥0.90. The isolated proteins were excluded from obtaining the protein-protein interaction network of *Panax notoginseng* for osteoporosis Fig. (**3**). Four hundred forty protein-protein interactions were obtained, presenting 880 relationships (edges). The obtained PPI network data were analyzed using the CytoNCA plug-in in Cytoscape software. The key target protein interaction maps of PN for osteoporosis were obtained by screening degree centrality Fig. (**4a**). Initially, nodes with degree centrality values above the median were selected Fig. (**4b**). Following this, a secondary screen was conducted, building upon the initial screening result and further refining the selection to include nodes with values greater than the median for each index Fig. (**4c**). Ultimately, this systematic approach led to the identification of key target proteins associated with PN for osteoporosis. Eventually, eight hub genes emerged: MAPK1, AKT1, RELA, JUN, FOS, TP53, HSP90AA1, and PPARA Fig. (**4c**). The configuration of MAPK1 protein product was depicted binding to quercetin in a 3D style, with a table indicating binding energies and minimal free energy Fig. (**5**).

### 3.4 GO and KEGG Analyses

GO and KEGG pathway analyses were conducted using the DAVID online database to investigate the 134 common genes of PN for osteoporosis. In GO enrichment, the biological process (BP) involves the response to xenobiotic stimulus, response to nutrient levels, response to oxidative stress, wound healing, cellular response to chemical stress, *etc.* The cellular component (CC) is mainly located in the membrane raft, membrane microdomain, transcription regulator complex, external side of plasma membrane, *etc.* The molecular function (MF) covers DNA-binding transcription factor binding, RNA polymeraseII-specific DNA-binding transcription factor binding, endopeptidase activity, and DNA-binding transcription activator, *etc.* Fig. (**6a**).

KEGG analyses yielded 163 enriched signaling pathways. The top 20 pathways related to osteoporosis are demonstrated in Fig. (**6b**), which principally comprise the AGE-RAGE signaling pathway in diabetic complications, TNF signaling pathway, IL-17 signaling pathway, p53 signaling pathway, NF-kappa B signaling pathway, PI3K-Akt signaling pathway, MAPK signaling pathway, FoxO signaling pathway, and Wnt signaling pathway, *etc.* These are the underlying signaling mechanisms of *Panax notoginseng* for osteoporosis.

To further elucidate the interconnections among these active ingredients, central genes, and signaling pathways, a Sankey diagram is utilized, as shown in Fig. (**7a**). Furthermore, Fig. (**7b**) employs network graph technology to illustrate the collaborative interaction of various signaling pathways as an integrated system in the treatment of osteoporosis.

### 3.5 Empirical Validation and Synergistic Research Insights

#### 3.5.1 Beta-Elemene

In recent years, there has been a growing emphasis on the role of inflammation and oxidative stress in the development of osteoporosis [1, 10]. Our search for literature on β-Elemene's role in combating osteoporosis did not reveal any studies dedicated to this specific area. Nonetheless, a plethora of research has established its anti-inflammatory and antioxidant effects, particularly in the field of cardiovascular therapy. The search results are presented in Table **1** [11-16].

#### 3.5.2 Quercetin

A substantial number of studies have been discovered focusing on the anti-osteoporotic effects of quercetin, indicating that it has been a hot topic of research. In-depth research in recent years has revealed many mechanisms by which it exerts its anti-osteoporotic effects, including the impact on the AMPK/SIRT1 signaling pathway, the regulation of autophagy and apoptosis, and the reduction of pro-inflammatory cytokines. The search results are presented in Table **2** [17-21].

#### 3.5.3 Methyl Palmitate

Methyl palmitate is a type of saturated fatty acid. This study focused on the relationship between palmitate and osteoporosis. Numerous *in vivo* and *in vitro* studies have indicated that it plays a promoting role in the pathogenesis of osteoporosis, including the suppression of osteogenic differentiation by increasing the expression of miR-92b-3p, affecting the β-catenin and Runx2/Smad signaling pathways during osteogenesis, and activating the NF-κB and ERK pathways to produce cytotoxicity and pro-inflammatory responses, among others. The relevant studies are listed in Table **3** [22-26].

#### 3.5.4 Oleic Acid

Oleic acid, a monounsaturated fatty acid, has been the subject of various investigations due to its potential impact on bone health. A wealth of research, both *in vivo* and in vitro, has suggested that oleic acid may have a protective effect against osteoporosis. This is attributed to its ability to modulate the expression of genes related to bone metabolism, such as the downregulation of sclerostin, which can enhance bone formation by inhibiting the Wnt signaling pathway. Additionally, oleic acid has been shown to reduce inflammation and oxidative stress, which are key factors in the development of osteoporosis. The detailed findings of these studies are compiled in Table **4** [27-30].

#### 3.5.5 Hexanal

Hexanal, a volatile aldehyde, has gained research attention for its potential role in combating osteoporosis. Several studies have demonstrated that hexanal could exert beneficial effects on bone health by stimulating osteoblast activity and inhibiting osteoclast function, thus promoting bone formation and reducing bone resorption. The mechanism by which hexanal influences bone health is not yet fully understood but may involve the activation of specific signaling pathways that regulate bone cell differentiation and function. A summary of the relevant research outcomes can be found in Table **5** [31-35].

## 4 DISCUSSION

Defined as the systemic deterioration of skeletal tissue, osteoporosis is captured by two essential characteristics: its emaciation effect on bone microstructure and bone mass and fragility fracture as the clinical outcome. Postmenopausal and aging-related bone loss are classified as primary osteoporosis. Secondary osteoporosis refers to specific pathological conditions leading to subsequent bone mass weakening, such as prolonged chemotherapy, excess glucocorticoid administration, and insufficient anabolic hormones following sharp weight loss [36]. Regardless of primary or secondary osteoporosis, the pathophysiological process can be succinctly summarized as the impairment of bone homeostasis balanced by osteoblasts-mediated bone matrix formation, and osteoclasts attributable bone resorption [37]. The bone homeostasis regulatory network involves a series of signaling pathways and numerous key regulatory nodes. A variety of anti-osteoporosis agents have been designed in the lab and emerged in clinical practice based on intricate modulatory mechanisms. Bisphosphonates, calcitonin, and selective estrogen receptor modulators are traditional prescriptions. Molecular-targeted agents, represented by denosumab and romosozumab, are getting broadly applied in clinical practice. Notwithstanding a range of osteoporosis agents, the treatment can be counterproductive since it occasionally results in the withholding of therapies by individuals due to side effects and poor compliance, especially in terms of sequential therapy, which requires regular patient follow-up [38]. As osteoporosis involves a tangled regulation network, it is primitive and far from desirable to rely on single-target medicine. In that light, it is crucial that we extend the pathogenesis research and seek a comprehensive mode of therapy.

In traditional Chinese medicine (TCM), the diagnosis incorporates inspection, olfaction, inquiry, and pulse examination to acquire holistic insights into humans. In parallel, the treatment process of TCM emphasizes the vitalistic and synthetic aspects of individuals. A typical Chinese herb or TCM formula contains abundant ingredients that synergize in the treatment of a disease, which enjoys greater efficacy and higher safety as compared to the application of a single ingredient, possibly attributable to the complementary effects, synergistic interactions, and mutual detoxification. TCM has been practiced to treat bone diseases, including osteopenia and osteoporosis, for hundreds of years [39]. Due to its global effect on versatile targets, TCM is receiving widespread attention and is regarded as the underlying comprehensive mode of therapy for osteoporosis. *Panax notoginseng*, which belongs to the family Araliaceae, plays a significant role in osteoporosis treatment [40]. In recent studies, it has been found that *Panax notoginseng* possesses the dual effect of promoting osteogenesis and inhibiting osteoclasts [41]. However, the mainstay of current studies is less than satisfactory for their fragmentation of *Panax notoginseng* into individual elements when studying its pharmaceutical effects, which is problematic in that it violates the holistic nature of the TCM by isolating some aspects from other influencing ingredients and thereby neglects the interactions among diverse components of the herbs. Decoding the anti-osteoporotic mechanisms of *Panax notoginseng* based on network pharmacology wisely avoids fragmentation and lopsidedness, closely and unbiasedly tracing out the authentic therapeutic effect of *Panax notoginseng*, drawing precise, enduring, and comprehensive conclusions that play guiding roles in further fundamental research and experimental verification.

It is generally believed that Western medicine is specific, microscopic, and scientific, while TCM is holistic, macroscopic, and dialectical [42]. Most noteworthy, it is becoming widely apparent across the globe that Western medicine and traditional Chinese Medicine are getting unified as growing evidence-based study methods are being applied to elucidate the mechanisms of TCM [43, 44]. Network pharmacology is one of the crucial bridges connecting evidence-based medicine and TCM. It reveals the associations between the therapeutic agents and the molecular targets based on the systemic level and biological network, taking into account the interactions among various constituents of the treatment scheme [45, 46], which coincides with the holistic and dialectical essence of TCM. Network pharmacology is widely used in the discovery of active compounds of TCM, the interpretation of the overall mechanism, and the analysis of TCM combinations and formula compatibility, *etc.*, providing new ideas for the study of complex systems of TCM and providing innovative scientific and technological support for rational clinical application and novel agents or formula research. In Western medicine, balancing the homeostasis between osteogenesis and osteoclasts is consonant with the TCM theoretical system, which pursues the balance between the *Yin* and *Yang* [47], emphasizing the harmonious coexistence of humanity with consideration of one’s age, gender, living circumstance, and dietary habit, *etc.*

We used TCMSP, GeneCards, TTD, PharmGkb, and OMIM databases to build interaction networks between compounds and disease targets. The results showed that *Panax notoginseng* intervenes in osteoporosis in a multi-systemic manner by affecting target genes, including MAPK1, AKT1, RELA, JUN, FOS, TP53, HSP90AA1, and PPARA, maintaining osteoblast-osteoclast balance and ameliorating bone metabolism. Concerning the hub genes from the PPI network depicting PN as an active ingredient for osteoporosis, According to reports in the literature, Mitogen-Activated Protein Kinase1 (MAPK1) is beneficial for anti-osteoporosis by promoting the proliferation and differentiation of osteoblasts, inducing the formation of vascular endothelial cells [48]. Jun ProtoOncogene (JUN) is closely related to the proliferation of osteoblasts and the differentiation, maturation, and activation of osteoclasts, in possession of a certain role in maintaining the dynamic balance between bone formation and bone resorption [49]. AKT Serine/Threonine Kinase 1 (AKT1) is a unique signaling vector in osteoblasts involved in controlling the differentiation of osteoblasts and osteoclasts [50]. Nuclear receptor PPARs act as transcription factors to control the expression of a large number of genes involved in metabolic homeostasis, lipid and glucose metabolism, adipogenesis, and inflammation. PPARs consist of three subtypes (α, β/σ, and γ). PPARA exerts a stimulatory effect on osteoblast differentiation of osteogenic precursor cells [51].

Moreover, GO enrichment analysis suggests that *Panax notoginseng*'s action on osteoporosis may be associated with immunity, inflammation, apoptosis, and other multi-BP processes. At the CC level, it is shown that the action of *Panax notoginseng* on osteoporosis covers cell membranes, cytoplasm, nuclei, transcription factor complexes, *etc.* From the MF level, *Panax notoginseng* may play a role in treating osteoporosis through cytokines, transcription factors, and other molecules. Previous studies have demonstrated that *Panax notoginseng* saponins(PNS) promote osteogenesis by enhancing cellular alkaline phosphatase activity, extracellular matrix mineralization, and the expression of osteoblast-associated molecules in osteoblasts [52]. PNS is also shown to stimulate the proliferation of bone marrow stromal cells and encourage their osteogenic differentiation through the upregulation of osteogenic marker genes and the downregulation of adipogenic marker genes in a dose-dependent manner [53]. An experimental team added active ingredients from *Panax notoginseng* to a bone tissue engineering scaffold, which was confirmed to promote angiogenesis and osteogenesis, increase osteoid tissue, and suppress osteoclast activity in osteoporotic bone defects [54].

*Panax notoginseng* can regulate processes such as metabolism, cell proliferation, cell survival, growth, and angiogenesis. The fundamental mechanism of osteoporosis is impaired bone resorption and bone production in the organism, so the signaling pathway related to bone metabolism is the keystone in the regulatory process. Regarding the results of KEGG pathway enrichment, it is noticeable that *Panax notoginseng* affects the relevant pathways of bone metabolism. PI3K/Akt signaling pathway regulates the proliferation, differentiation, and apoptosis of osteoblasts and osteoclasts [55]. If the PI3K/Akt signaling pathway can be regulated to modulate the function of osteoblasts and osteoclasts, the balance between osteoblasts and osteoclasts can be maintained, avoiding the occurrence of bone homeostasis imbalance [56]. Interleukin-17 is involved in the pathogenesis of osteoporosis by affecting bone density [57, 58]. Wild-type mice undergoing oophorectomy suffer from trabecular osteoporosis due to estrogen deletion, while mice with missing interleukin-17 receptors have a complete trabecula [57]. In clinical trials, the higher the interleukin-17 in the serum of postmenopausal osteoporosis patients, the lower the bone density [59]. The inflammatory response mediated by the interleukin-17 signaling pathway has an important regulatory role in the pathophysiological process of rheumatoid arthritis complicated by osteoporosis, and its mechanism is mainly that interleukin-17 inhibits the repair effect of proteoglycans and collagen on the matrix, thereby destroying extracellular matrix and cartilage synthesis [60].

This study analyzed the potential mechanism of *Panax notoginseng* in treating osteoporosis from the perspective of network pharmacology and identified the main active drugs and different central genes and signaling pathways related to bone formation. However, this study only included bioinformatics analysis and needs to be supported by corresponding *in vitro* and *in vivo* experimental observations. This study is exploratory and increases our understanding of the antagonistic effect of *Panax notoginseng* on osteoporosis. It is worthwhile further to study its active components, targets, and action networks.

## CONCLUSION

From the network pharmacological analysis, it can be obtained that *Panax notoginseng* intervenes in osteoporosis in a multi-systemic manner by affecting target genes including MAPK1, AKT1, RELA, JUN, FOS, TP53, HSP90AA1, and PPARA, regulating signaling pathways inclusive of IL-17, p53, NF-kappa B, PI3K-Akt, MAPK, FoxO, and Wnt signaling pathway, maintaining osteoblast-osteoclast balance and ameliorating bone metabolism, which firmly coincides with the holistic concept of Traditional Chinses Medicine. This study elucidated the mechanism of *Panax notoginseng* for osteoporosis, but further experimental validation of its active ingredients, targets, and network of action is required to provide sufficient evidence for clinical application. *Panax notoginseng* belongs to the ginseng species. The common ginsengs are Panax ginseng (PG, or Korean ginseng), *Panax notoginseng* (PN, or Chinese ginseng), Panax quinquefolium (PQ, or North American ginseng), and Panax japonicus (PJ, or Japanese ginseng) [40]. We believe that our work will provide broader insights for novel research and developments of the Ginseng species for osteoporosis.

## Figures and Tables

**Fig. (1):**
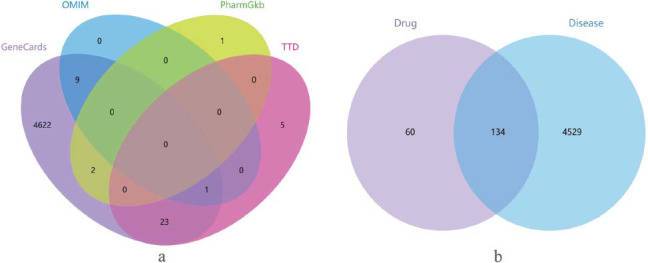
Venn of osteoporosis targets from four databases and the intersection of PN and osteoporosis. (**a**): GeneCards, OMIM, PharmGkb, and TTD databases were searched for the collection of osteoporosis-related genes. In Fig. (**1a**), the color purple, blue, yellow, and red correspond to GeneCards, TTD, PharmGkb, and OMIM databases. The number in each color region represents the number of genes retrieved in the corresponding database. The number in the overlapping area is the same genes retrieved from different databases. (**b**): 134 targets were related to both PN and osteoporosis.

**Fig. (2):**
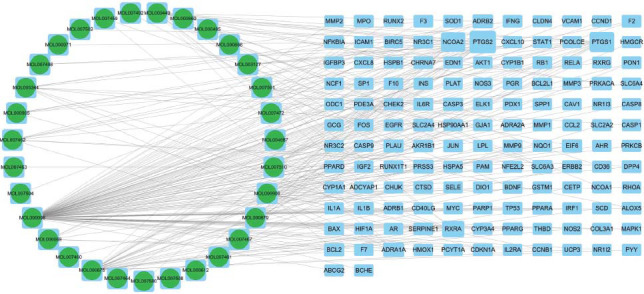
Interaction network between putative medicinal ingredients of PN (circular portions on the left half) and putative disease targets of osteoporosis (matrix portions on the right half).

**Fig. (3):**
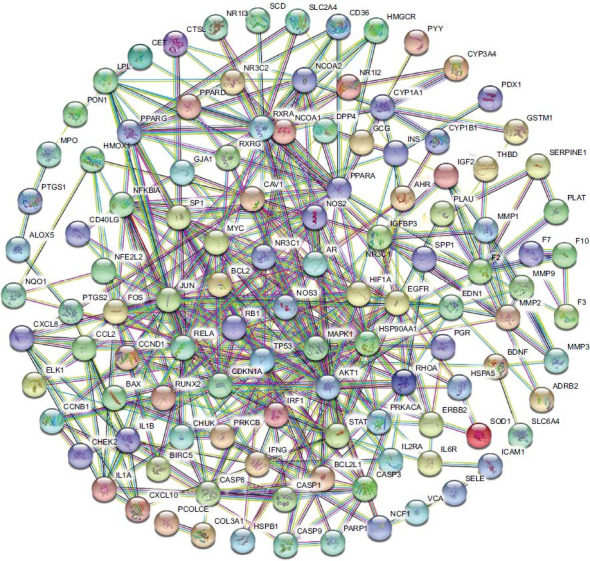
PPI network about osteoporosis-related targets. Nodes: Network nodes represent proteins. Node Color: colored nodes: query proteins and first shell of interactors; white nodes: second shell of interactors. Node Content: empty nodes: proteins of unknown 3D structure; filled nodes: a 3D structure is known or predicted. Edges: Edges represent protein-protein associations. The significance of edges with different colors can be obtained on the String database (https://cn.string-db.org/) or refer to the supplementary materials.

**Fig. (4):**
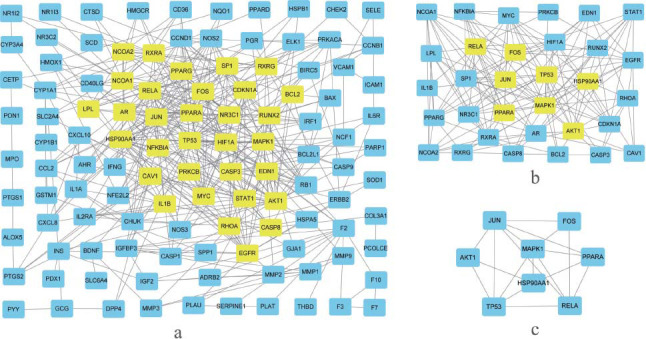
Centrality selection of putative disease targets of osteoporosis. (**a**): the original network of putative disease targets. (**b**): central genes after one round of centrality limitation. (**c**): Hub genes after the second round of centrality calculation.

**Fig. (5):**
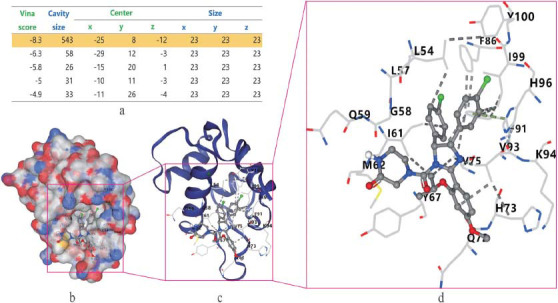
Demonstration of MAPK1 binding with quercetin in the lowest free energy style. (**a**): binding energies table with the fitness value. (**b**): binding pattern with surface covering. (**c**): naked three-dimensional binding pattern. (**d**): specific amino acids with their potential binding sites.

**Fig. (6):**
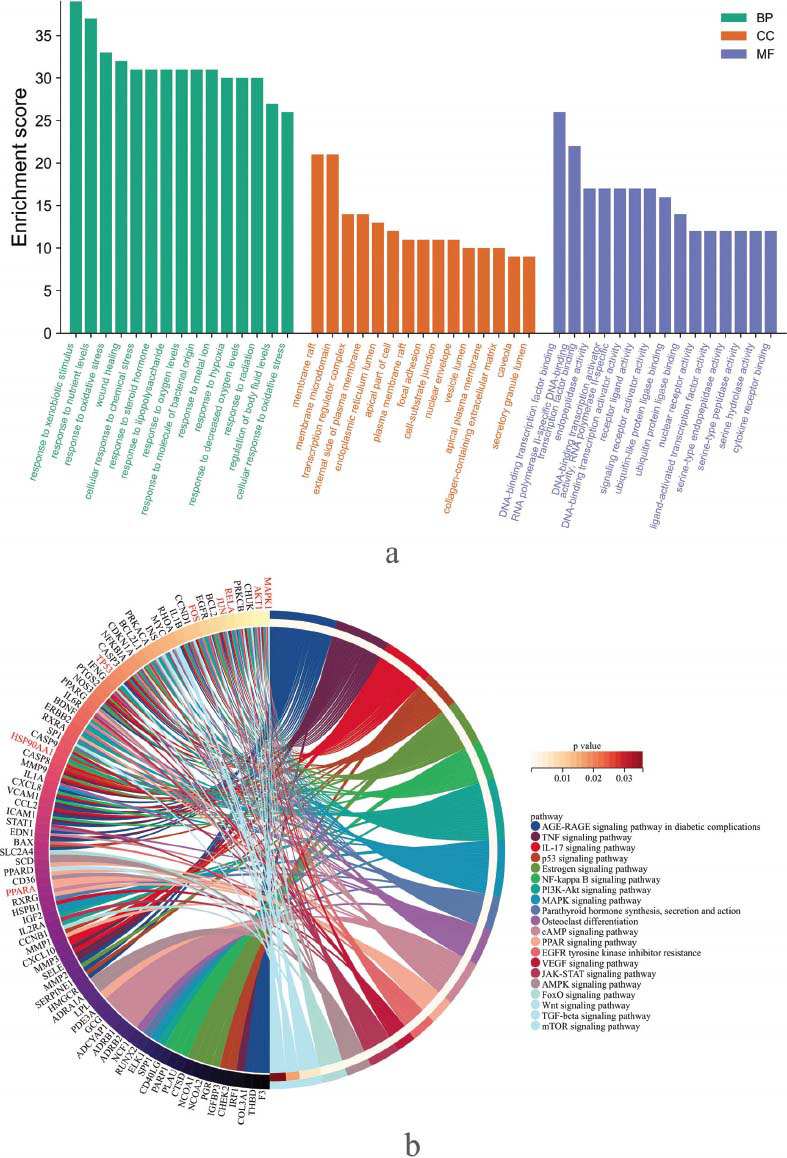
GO and KEGG analyses. (**a**): bar plot demonstrating biological process (BP), cellular component (CC), and molecular function (MF) of GO function analyses. (**b**): Circos demonstrating KEGG pathway analyses. The top pathways enriching the most genes were presented.

**Fig. (7):**
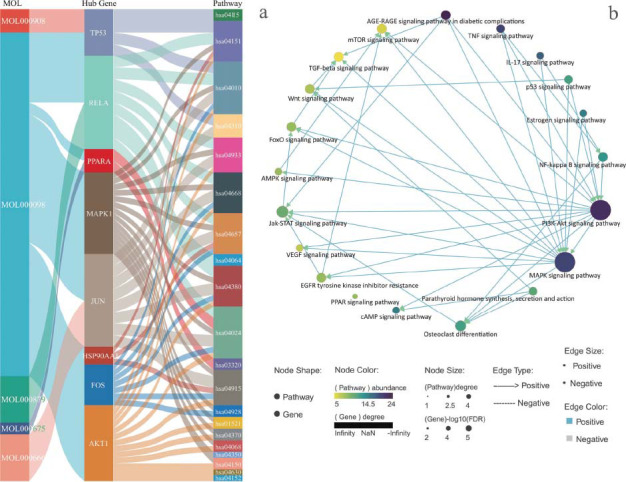
Interactions among PN ingredients, hub genes, and signaling pathways. (**a**): Sankey diagram demonstrating the relationships among PN ingredients, hub genes, and signaling pathways. (**b**): Network among osteoporosis-related signaling pathways. MOL000908: beta-elemene. MOL000098: quercetin. MOL000879: methyl palmitate. MOL000675: oleic acid. MOL000666: hexanal.

**Table 1:** Beta-elemene.

**No.**	**Article**	**Country**	**Design**	**Study Models**	**Objectives**	**Main Results**
1	Patra, *et al.,* (2016) [11]	Saint Lucia	Experimental study with *in vivo* and *in vitro* models	Carrageenan-induced paw edema, Cotton pellet granuloma, Acetic acid-induced vascular permeability, LPS-induced murine macrophage model	The study objectives are to evaluate the therapeutic potential and anti-inflammatory activity of Delonix regia ethanol extract and β-Elemene, determine their effects on inflammatory mediators and signaling pathways, and assess their safety in terms of acute and sub-acute toxicity.	Delonix regia extract and β-Elemene significantly inhibit inflammatory cytokines and signaling pathways, with no observed toxicity, and β-Elemene exhibits potential as an anti-inflammatory agent through its strong binding affinity to COX-II.
2	Fang, *et al.,* (2018) [12]	China	Experimental study with *in vitro* models	Lipopolysaccharide (LPS)-induced murine macrophage cells RAW264.7	To investigate the anti-inflammatory effects and potential molecular mechanisms of β-elemene in macrophage activation	β-elemene significantly suppressed the production of pro-inflammatory mediators (IL-6, TNF-α, IL-1β) in a dose-dependent manner. It also inhibited iNOS and IL-10 expression related to the down-regulation of the Wnt/β-catenin signaling pathway.
3	Chen, *et al.,* (2015) [13]	China	Experimental study with *in vitro* models	Human Umbilical Vein Endothelial Cells (HUVECs) treated with H_2_O_2_	To investigate the antioxidant activity of synthesized 13-β-elemene ester derivatives	Derivatives 5v and 5w of β-elemene exerted potent antioxidant effects, significantly attenuating ROS induction in H_2_O_2_-treated HUVECs. They efficaciously modulated the expression of SOD and MDA, and mitigated the oxidative stress-mediated increase in NO and LDH levels. The compounds demonstrated no cytotoxicity and enhanced cell viability in a dose-dependent manner.
4	Liu, *et al.,* (2017) [14]	China	Experimental study with *in vitro* and *in vivo* models	ApoE−/− mice, Human Umbilical Vein Endothelial Cells (HUVECs)	To investigate the effects of β-elemene on atherosclerotic plaques area and endothelial dysfunction in ApoE−/− mice, and its effects on phosphorylation of eNOS and Akt in HUVECs	β-elemene reduced aortic lipid peroxidation, as indicated by decreased plasma and aortic F2-isoprostanes levels. Additionally, β-elemene increased the activity of antioxidant enzymes like superoxide dismutase, catalase, and glutathione peroxidase in the aortic tissue. The treatment also downregulated the expression of inflammatory markers in the aorta, suggesting its anti-inflammatory effect.
5	Ahmad, *et al.,* (2018) [15]	China	Experimental study with *in vitro* models	Human Umbilical Vein Endothelial Cells (HUVECs) treated with H_2_O_2_	To evaluate the antioxidant activity and the underlying mechanisms of a novel β-elemene derivative, Bis(β-elemene-13-yl), glutarate (BEG), in protecting HUVECs from oxidative stress	BEG significantly reduced H_2_O_2_-induced cell viability loss, ROS production, LDH release, and MDA levels in a concentration-dependent manner. BEG increased SOD activity and upregulated the PI3K/Akt/eNOS/NO pathway, which was inhibited by a PI3K inhibitor (wortmannin) and eNOS inhibitor (L-NAME).
6	Liu, *et al.,* (2015) [16]	China	Experimental study with *in vitro* models	Human umbilical vein endothelial cells (HUVECs) and human peripheral blood monocyte (THP-1) cells	To investigate the effects of β-elemene on monocyte–endothelial cells interactions in the initiation of atherosclerosis	β-elemene protects HUVECs from H_2_O_2_-induced injury and inhibits monocyte adhesion and transendothelial migration. It decreases ROS generation, prevents MAPK activation, and suppresses NF-κB-dependent expression of cell adhesion molecules.

**Table 2:** Quercetin.

**No.**	**Article**	**Country**	**Design**	**Study Models**	**Objectives**	**Main Results**
1	Li, *et al.,* (2024) [17]	China	Experimental study with *in vitro* and *in vivo* models	*In vitro*: Mouse Mesenchymal Stem Cells *In vivo*: Zebrafish model of dexamethasone-induced osteoporosis	To investigate the potential of chitosan-quercetin bio-conjugate as an anti-osteoporotic agent	Chitosan-quercetin bio-conjugate enhanced osteogenic differentiation markers, increased calcium deposition in MSCs, and improved bone calcification and callus formation in zebrafish models. It also inhibited osteoclastic activity, suggesting its effectiveness in mitigating bone resorption.
2	Wang, *et al.,* (2023) [18]	China	Experimental study with *in vivo* and *in vitro* models	*In vitro*: Mouse Mesenchymal Stem Cells *In vivo*: Middle-aged female rats	To investigate the therapeutic effects of dasatinib and quercetin combination on postmenopausal osteoporosis and bone regeneration	Systemic treatment with dasatinib and quercetin (DQ) ameliorated postmenopausal osteoporosis in middle-aged rats by reducing senescent cell accumulation and restoring MSC function. Local administration of DQ in combination with BMP2 enhanced osteogenic differentiation of MSCs and improved bone regeneration in osteoporotic conditions.
3	Vakili, *et al.,* (2021) [19]	Iran	Experimental study with *in vivo* models	Ovariectomized rats treated with Quercetin and/or vitamin E	To investigate the effects of quercetin and vitamin E on ovariectomy-induced osteoporosis and their potential mechanisms through modulating autophagy and apoptosis	Treatment with quercetin and vitE markedly improved osteoporosis by reversing the ovariectomy-induced changes in serum calcium, bone weight, bone volume, and the total number of osteocytes and osteoblasts. It also decreased the total number of osteoclasts and serum osteocalcin. Furthermore, Q and vitE regulated the mRNA expressions of autophagy-related genes (LC3, beclin1) and apoptosis-related genes (caspase 3, bcl2) in the tibia, suggesting their role in preventing osteoporosis by modulating these processes.
4	Feng, *et al.,* (2024) [20]	China	Experimental study with *in vivo* models	Ovariectomized (OVX) rats treated with quercetin, with and without antibiotic treatment, and fecal microbiota transplantation (FMT)	To investigate the effects of quercetin on the “intestinal flora - short-chain fatty acids (SCFAs) - inflammatory” signaling axis in ovariectomized rats and its potential as a treatment for osteoporosis	Quercetin increased potentially probiotic bacteria and decreased potentially pathogenic bacteria. It also increased SCFAs content, enhanced tight junction proteins, and reduced proinflammatory cytokine levels (LPS, IL-1β, TNF-α), leading to improved bone strength and prevention of OVX-induced bone loss.
5	Wang, *et al.,* (2021) [21]	China	Experimental study with *in vitro* models	Mouse bone mesenchymal stem cells	To investigate the effects of quercetin on the osteogenic differentiation and antioxidant responses of mBMSCs, and to explore its underlying mechanism	Quercetin treatment induced significant upregulation of antioxidant enzymes (SOD1 and SOD2) in mBMSCs. It promoted osteogenic differentiation and inhibited adipogenic differentiation. Quercetin enhanced the phosphorylation of AMPK protein and upregulated the expression of SIRT1, activating the AMPK/SIRT1 signaling pathway in mBMSCs.

**Table 3:** Methyl palmitate.

**No.**	**Article**	**Country**	**Design**	**Study Models**	**Objectives**	**Main Results**
1	Wang, *et al.,* (2023) [22]	China	Experimental study with *in vivo* and *in vitro* models	*In vitro*: MC3T3-E1 cells. *In vivo*: Rat osteoporotic model post-ovariectomy	To explore the role of fatty acids in the early stages of postmenopausal osteoporosis and their influence on osteogenic differentiation through microRNAs	Palmitate showed suppressive effects on osteogenic differentiation by increasing miR-92b-3p expression, which in turn inhibits osteoblastogenesis through the PTEN pathway.
2	Gunaratnam, *et al.,* (2014) [23]	USA	Experimental study with *in vitro* models	Human osteoblasts (Obs)	To characterize the lipotoxic effect of palmitate on human osteoblasts and explore its mechanisms on bone cell function and differentiation	Palmitate negatively affected differentiation and bone nodule formation and mineralization by osteoblasts. It induced significant alterations in the β-catenin and Runx2/Smad signaling pathways, which play crucial roles in osteogenesis.
3	Gillet, *et al.,* (2015) [24]	Belgium	Experimental study with *in vitro* models	Human mesenchymal stem cells (MSC) and MSC-derived osteoblastic cells	To examine the effect of palmitate and oleate on the function and survival of human MSC and osteoblastic cells	Palmitate induced cytotoxicity and a proinflammatory response in MSC and osteoblastic cells, while oleate neutralized these effects. Palmitate triggered endoplasmic reticulum stress and activation of NF-κB and ERK pathways. Oleate prevented palmitate-induced lipotoxicity by inhibiting these pathways and promoting palmitate esterification into triglycerides and storage in lipid droplets.
4	Tao, *et al.,* (2024) [25]	China	Experimental study with *in vivo* and *in vitro* models	*In vitro*: MC3T3-E1 cells, RAW264.7 cells *In vivo*: Ovariectomized (OVX) rats	To evaluate the effects of Astaxanthin (ATX) on methyl palmitate (palmitic acid)-induced bone loss and cellular oxidative stress	Palmitic acid treatment resulted in decreased bone mass and bone mineral density, and increased oxidative stress in OVX rats. In MC3T3-E1 cells, palmitic acid impaired osteogenic differentiation, reduced alkaline phosphatase expression, and inhibited calcified nodule formation. Palmitic acid stimulated osteoclast differentiation in RAW264.7 cells, as indicated by increased TRAP formation.
5	Guo, *et al.,* (2022) [26]	China	Experimental study with *in vivo* and *in vitro* models	*In vivo*: High-fat diet (HFD)-fed obese mice In vitro: MC3T3-E1 cells treated with palmitic acid	To investigate the protective effects of FGF19 on high-fat diet (HFD)-fed obese mice and palmitic acid (PA)-treated osteoblasts	Palmitic acid treatment resulted in decreased bone mineral density and bone mass in high-fat diet fed obese mice. In MC3T3-E1 cells, palmitic acid impaired osteogenic differentiation, reduced ALP activity, and decreased protein expression of Collagen-1, Runx-2, and osteopontin. Palmitic acid increased cell death and negatively affected cell morphology in osteoblasts.

**Table 4:** Oleic acid.

**No.**	**Article**	**Country**	**Design**	**Study Models**	**Objectives**	**Main Results**
1	Kasonga, *et al.,* (2019) [27]	South Africa	Experimental study with *in vitro* models	*In vitro*: RAW264.7 murine macrophages (pre-osteoclasts) and MC3T3-E1 murine pre-osteoblasts	To determine the role of FFAR4 in the action of UFAs in bone cells and to elucidate the mechanism of action of oleic acid in bone	Oleic acid inhibited RANKL-induced osteoclast differentiation in RAW264.7 cells in an FFAR4-dependent manner, reducing the formation of TAK1-TAB1 complexes and activation of NF-κB and MAPK signaling pathways. In MC3T3-E1 cells, oleic acid enhanced osteoblast signaling through the FFAR4/βarr2 axis, increasing ALP activity and upregulating the expression of osteoblast-specific genes such as Runx2, COL1A1, and BSP, while also increasing the OPG/RANKL ratio, thus inhibiting osteoclast formation.
2	Schaepe, *et al.,* (2017) [29]	Germany	Experimental study with *in vitro* models	Human mesenchymal stromal cells (hMSCs) from osteoporotic and non-osteoporotic donors	To study the fatty acid composition of the cell plasma membranes during osteogenic and adipogenic differentiation and its relation to osteoporosis	In osteogenic differentiation, oleic acid (FA (18:1)) showed increased relative mass signal intensities in non-osteoporotic cells compared to osteoporotic cells, suggesting a role in promoting osteogenic differentiation.
3	Fonolla-Joya, *et al.,* (2016) [30]	Spain	Clinical intervention study	Postmenopausal women	To evaluate the effects of a dairy product enriched with polyunsaturated fatty acids, calcium, oleic acid, and vitamins on cardiovascular markers and bone metabolism in postmenopausal women with moderate cardiovascular risk	In the intervention group, there was a significant decrease in the levels of high-sensitivity C-reactive protein (hs-CRP) and a reduction in the receptor activator of nuclear factor kB ligand (RANKL), which is associated with decreased bone resorption. These findings suggest that the enriched milk product may have a positive impact on bone metabolism, potentially beneficial for preventing osteoporosis.

**Table 5:** Hexanal.

**No.**	**Article**	**Country**	**Design**	**Study Models**	**Objectives**	**Main Results**
1	Lambertini, *et al.,* (2021) [31]	Italy	Experimental study with *in vitro* models	Human primary osteoblasts and osteoclasts	To investigate the potential pro-osteogenic effects of hexane, acetone, and methanol extracts of Cucurbita moschata leaves, particularly the effects of hexanal-related compound PU-13OH-FA	The acetone extract, which contains the hexanal-related compound PU-13OH-FA, showed potential in stimulating osteoblast function and inhibiting osteoclast differentiation. This suggests that these extracts could help manage metabolic bone disorders such as osteoporosis and promote tissue healing after bone fractures.
2	Chaugule, *et al.,* (2019) [32]	India	Experimental study with *in vitro* and *in vivo* models	*In vitro*: Mouse bone marrow-derived macrophages (BMMs) *In vivo*: Ovariectomized (OVX) Swiss mice	To evaluate the anti-osteoporotic effect of hexane fraction of Turbo brunneus methanolic extract (HxTME) and its role in RANK-RANKL signaling pathway	HxTME significantly inhibited RANKL-induced osteoclast differentiation and maturation *in vitro*. It downregulated key transcription factors and osteoclast-related genes. *In vivo*, HxTME preserved bone microarchitecture and mineral content, inhibiting bone loss in OVX mice.
3	Pathomwichaiwat, *et al.,* (2015) [33]	Thailand	Experimental study with *in vitro* models	MC3T3-E1 osteoblastic cells	To identify active compounds in Cissus quadrangularis hexane extract that stimulate osteoblast differentiation	A total of 29 compounds were isolated, including triterpenes, fatty acid methyl esters, glycerolipids, steroids, phytols, and cerebrosides. Four new dammarane-type triterpenes were identified, and seven compounds stimulated ALP activity, indicating a role in osteoblast differentiation. The study suggests that hexanal-related compounds in Cissus quadrangularis hexane extract may contribute to the anti-osteoporotic effects of the plant.
4	Kim, *et al.,* (2011) [34]	Korea	Experimental study with *in vitro* and *in vivo* models	*In vitro*: Osteoblastic cell lines C3H10T1/2 and MC3T3-E1 *In vivo*: Male ICR mice model	To evaluate the inhibitory effects of hexane extract of Poncirus trifoliata (PT-H) on glucocorticoid-induced osteoporosis (GIO) and to identify its molecular mechanisms	PT-H inhibited Dex-induced apoptosis in osteoblastic cells and increased bone mineral density (BMD) in GIO mice model. The extract decreased the expression of AnxA6, which may play a key role in the inhibition of GIO.
5	Phromnoi, *et al.,* (2022) [35]	Thailand	Experimental study with *in vitro* models	Mouse macrophage RAW264.7 cells, human osteoblast-like MG-63 and SAOS-2 cells	To investigate the effects of Perilla frutescens leaf hexane fraction (PLH), rich in luteolin and baicalein, on osteoclast and osteoblast activity and differentiation	PLH inhibited RANKL-induced ROS production, TRAP-positive multinucleated osteoclasts, and downregulated MAPK and NF-κB signaling pathways. Additionally, PLH enhanced osteoblast function by increasing ALP activity and restored TNF-α-suppressed osteoblast proliferation and osteogenic potential.

## Data Availability

All data included in this study are available upon request by contact with the corresponding author.

## LIST OF ABBREVIATIONS

BMD: Bone Mineral Density
CC: Cellular Component
OB: Oral Bioavailability
DL: Drug-Likeness
MF: Molecular Function
PN: Panax Notoginseng
PPI: Protein-Protein Interaction
SERM: Selective Estrogen Receptor Modulator
