# Supplementary material for: Network Pharmacology Unveils Multi-Systemic Intervention of Panax notoginseng in Osteoporosis via Key Genes and Signaling Pathways
Source: Endocr Metab Immune Disord Drug Targets. 2025 Jan 9;25(15):1211–27. doi: 10.2174/0118715303335018241107084224 (PMC12709535; doi:10.2174/0118715303335018241107084224)
Supplement: Supplementary file 1 [file EMIDDT-25-15-1211_SD1.pdf]

Supplementary Material

Network Pharmacology Unveils Multi-Systemic Intervention of Panax notoginseng in Osteoporosis *via* Key Genes and Signaling Pathways

Qiyue Wang[1], Xiaoping Wang[2], Kezhou Wu[1,*], Weiwei Wu[2], Zhantu Wei[2] and Weili Feng[2,*]

[1] Sports Medicine Center, Department of Orthopaedic Surgery, The First Affiliated Hospital of Shantou University Medical College, Shantou, Guangdong Province, People's Republic of China; [2]Department of Orthopaedics, Xiaolan People's Hospital of Zhongshan, Zhongshan, Guangdong Province, People's Republic of China

Table 1. TCMSP-derived OB and DL characteristics of 86 ingredients in PN.

No.	Molecular ID	Molecular Name	Oral Bioavailability	Drug Likeness
1	MOL004464	MEHQ	43.98149625	0.019294
2	MOL000449	Stigmasterol	43.82985158	0.75665
3	MOL004687	2-octanone	19.28634805	0.011434
4	MOL000485	TMH	46.24981501	0.052565
5	MOL005284	(3S,5R,6S,8R,9R,10R,12R,13R,14R,17S)-17-[(2S)-2-hydroxy-6-methylhept-5-en-2-yl]-4,4,8,10,14-pentamethyl-2,3,5,6,7,9,11,12,13,15,16,17-dodecahydro-1H-cyclopenta[a]phenanthrene-3,6,12-triol	20.12704418	0.77961
6	MOL005319	Ditertbutyl phthalate	43.66867352	0.12938
7	MOL005334	(3S,5R,8R,9R,10R,12R,13R,14R,17S)-17-[(2S)-2-hydroxy-6-methylhept-5-en-2-yl]-4,4,8,10,14-pentamethyl-2,3,5,6,7,9,11,12,13,15,16,17-dodecahydro-1H-cyclopenta[a]phenanthrene-3,12-diol	29.69203747	0.77048
8	MOL005337	(2S,3R,4S,5S,6R)-2-[(2S)-2-[(3S,5R,8R,9R,10R,12R,13R,14R,17S)-3-[(2R,3R,4S,5S,6R)-4,5-dihydroxy-6-(hydroxymethyl)-3-[(2S,3R,4S,5S,6R)-3,4,5-trihydroxy-6-(hydroxymethyl)oxan-2-yl]oxyoxan-2-yl]oxy-12-hydroxy-4,4,8,10,14-pentamethyl-2,3,5,6,7,9,11,12,13,15,1	5.499218806	0.094758
9	MOL005338	Ginsenoside Re	4.272725573	0.12287
10	MOL005340	(3S,5R,6S,8R,9R,10R,12R,13R,14R,17S)-17-[(2R)-2-hydroxy-6-methylhept-5-en-2-yl]-4,4,8,10,14-pentamethyl-2,3,5,6,7,9,11,12,13,15,16,17-dodecahydro-1H-cyclopenta[a]phenanthrene-3,6,12-triol	20.12704418	0.77985
11	MOL005341	Sanchinoside C1	10.04245744	0.27807

12	MOL005344	ginsenoside rh2	36.31951162	0.55868
13	MOL000908	beta-elemene	25.63362343	0.060519
14	MOL005538	Linolenyl alcohol	42.79174531	0.11781
15	MOL005843	1-Hydroxycumene	59.9735836	0.02573
16	MOL000612	(-)-alpha-cedrene	55.56099313	0.10498
17	MOL000066	alloaromadedrene	53.46135969	0.10414
18	MOL000666	hexanal	55.70702938	0.0055795
19	MOL006728	Gypenoside XIV_qt	29.69203747	0.77174
20	MOL000675	oleic acid	33.12836481	0.14243
21	MOL000069	palmitic acid	19.2965647	0.098573
22	MOL000705	WLN: VH6	19.5879957	0.0080592
23	MOL000711	2,4-Heptdienal	19.13815202	0.0085495
24	MOL007459	[(1R)-1-methoxyethyl]benzene	42.09107328	0.022299
25	MOL007460	Cyclooctadiene	42.82230102	0.010757
26	MOL007461	1,2-DIHYDRO-1,5,8-TRIMETHYLNAPHTHALENE	47.68809786	0.059783
27	MOL007462	1,4,6-trimethyl-1,2,3,4-tetralin	45.15023343	0.058276
28	MOL007463	(1R,2S)-1-ethyl-2-methylcyclopropane	39.60135081	0.010807
29	MOL007464	1-methyl-5-isopropenyl cyclohexene	48.12159348	0.022412
30	MOL007465	10-Methylnonadecane	10.28466145	0.12067
31	MOL007466	DICHLOROANILINE	44.51332132	0.020203
32	MOL007467	2,6-dimethyl-cyclohexanol	76.2833927	0.020172
33	MOL007468	3,5-Dimethoxyacetophenone	27.61340737	0.044445
34	MOL007469	3,4-dichloroaniline	9.919162383	0.019111
35	MOL007470	(2E)-3-ethylpenta-2,4-dien-1-ol	27.1293882	0.0091687
36	MOL007471	Octadecyne	4.65903529	0.093941
37	MOL007472	(9Z,12E)-octadeca-9,12-dienoic acid methyl ester	41.93435814	0.16705
38	MOL007473	N3-Oxalyl-L-2,3-diaminopropanoate	72.92559815	0.036444

39	MOL007474	(2R,3S,4S,5R,6S)-2-(hydroxymethyl)-6-[(2S)-6-methyl-2-[(3S,5R,6S,8R,9R,10R,12R,13R,14R,17S)-3,6,12-trihydroxy-4,4,8,10,14-pentamethyl-2,3,5,6,7,9,11,12,13,15,16,17-dodecahydro-1H-cyclopenta[a]phenanthren-17-yl]hept-5-en-2-yl]oxyoxane-3,4,5-triol	4.05373169	0.59722
40	MOL007475	ginsenoside f2	36.43174722	0.25282
41	MOL007476	ginsenoside rb1	6.287543461	0.038132
42	MOL007477	(2S,3R,4S,5S,6R)-2-[(2S)-2-[(3S,5R,8R,9R,10R,12R,13R,14R,17S)-3-[(2R,3R,4S,5S,6R)-4,5-dihydroxy-6-(hydroxymethyl)-3-[(2S,3R,4S,5S,6R)-3,4,5-trihydroxy-6-(hydroxymethyl)oxan-2-yl]oxyoxan-2-yl]oxy-12-hydroxy-4,4,8,10,14-pentamethyl-2,3,5,6,7,9,11,12,13,15,1	6.022169847	0.043359
43	MOL007478	ginsenoside rb3	7.841533461	0.043355
44	MOL007479	ginsenoside rc	8.12585372	0.043566
45	MOL007480	ginsenoside rd_qt	12.22587638	0.77439
46	MOL007481	ginsenoside rf	15.32745324	0.24165
47	MOL007482	(2S,3R,4R,5R,6S)-2-[[(2R,3R,4S,5S,6R)-2-[[(3S,5R,6S,8R,9R,10R,12R,13R,14R,17S)-3,12-dihydroxy-17-[(1S)-1-hydroxy-1,5-dimethylhex-4-enyl]-4,4,8,10,14-pentamethyl-2,3,5,6,7,9,11,12,13,15,16,17-dodecahydro-1H-cyclopenta[a]phenanthren-6-yl]oxy]-4,5-dihydroxy-	10.09229647	0.25818
48	MOL007483	ginsenoside rg3	5.946658515	0.043637
49	MOL007484	(2R,3R,4S,5S,6R)-2-[[(3S,5R,6S,8R,9R,10R,12R,13R,14R,17S)-3,12-dihydroxy-17-[(2S)-2-hydroxy-6-methylhept-5-en-2-yl]-4,4,8,10,14-pentamethyl-2,3,5,6,7,9,11,12,13,15,16,17-dodecahydro-1H-cyclopenta[a]phenanthren-6-yl]oxy]-6-(hydroxymethyl)oxane-3,4,5-triol	16.90791283	0.57129
50	MOL007485	(2S,3S,4S,5R,6R)-6-[[(3S,4aR,6aR,6bS,8aS,12aS,14aR,14bR)-4,4,6a,6b,11,11,14b-heptamethyl-8a-[oxo-[[(2S,3R,4S,5S,6R)-3,4,5-trihydroxy-6-(hydroxymethyl)-2-tetrahydropyranyl]oxy]methyl]-1,2,3,4a,5,6,7,8,9,10,12,12a,14,14a-tetradecahydropicen-3-yl]oxy]-3,4-di	1.950387927	0.054962
51	MOL007486	ZINC01532096	50.73994592	0.14658
52	MOL007487	notoginsenosider1	5.42875197	0.1311
53	MOL007488	notoginsenosider2	7.691339823	0.27634
54	MOL007489	notoginsenosider3	3.013720692	0.038121
55	MOL007490	notoginsenosider3_qt	13.83247838	0.77362

56	MOL007491	notoginsenosider4	7.345155594	0.017043
57	MOL007492	Butylcyclobutane	47.58718335	0.014749
58	MOL007493	(1S,4S,4aS,6S,8aS)-4-isopropyl-1,6-dimethyldecahydronaphthalene	23.2916363	0.076257
59	MOL007494	gypenosideix	8.81950072	0.11492
60	MOL007495	gypenosidexvii	4.864340863	0.10072
61	MOL007496	isopulegone	55.39492772	0.030953
62	MOL007497	NaPst	46.9256179	0.017427
63	MOL007498	10Z,13Z-nonadecadienoic acid	40.9826465	0.17009
64	MOL013377	Lutein	22.58655524	0.54529
65	MOL007500	panaxatriol	15.41984471	0.79324
66	MOL007501	panaxydol	61.66659941	0.13157
67	MOL007502	NSC692928	43.31317299	0.098896
68	MOL007503	panaxytriol	33.75825582	0.12863
69	MOL007504	NSC 308879	17.1403624	0.79515
70	MOL007505	WLN: QR DG	60.43659664	0.014552
71	MOL007506	protopanoxadiol	21.14073099	0.78312
72	MOL007507	sanchinan-a	7.571344374	0.027322
73	MOL007508	α-cyperene	51.10460078	0.10893
74	MOL007509	(4aS,9aR)-2,9,9-trimethyl-5-methylene-4,4a,6,7,8,9a-hexahydro-3H-benzo[7]annulene	22.73526392	0.079885
75	MOL007510	α-copaene	37.81377189	0.079983
76	MOL007511	(5S)-5-ethyloxolan-2-one	75.6885177	0.013646
77	MOL000860	stearic acid	17.82542938	0.14086
78	MOL000864	MYS	13.9810568	0.04922
79	MOL000867	Heptadekan	8.642472374	0.074658
80	MOL000869	Henicosane	8.412905294	0.15364
81	MOL000879	methyl palmitate	18.08756063	0.11594
82	MOL000905	()-beta-Pinene	44.76823783	0.052595

83	MOL003127	Germacrene D	19.22250577	0.057074
84	MOL000935	Hepanal	53.83317567	0.10397
85	MOL000971	Ethylpalmitate	18.98672237	0.13539
86	MOL000098	quercetin	46.43334812	0.27525

Table 2. PN Ingredients and Corresponding Targets.

No.	Molecluar Id	Molecular Name	Target
1	MOL004464	MEHQ	Lysozyme
2	MOL004464	MEHQ	Bacillolysin
3	MOL004464	MEHQ	Nicotinate-nucleotide--dimethylbenzimidazole phosphoribosyl-transferase
4	MOL000449	Stigmasterol	Progesterone receptor
5	MOL000449	Stigmasterol	Mineralocorticoid receptor
6	MOL000449	Stigmasterol	Nuclear receptor coactivator 2
7	MOL000449	Stigmasterol	Ig gamma-1 chain C region
8	MOL000449	Stigmasterol	Retinoic acid receptor RXR-alpha
9	MOL000449	Stigmasterol	Nuclear receptor coactivator 1
10	MOL000449	Stigmasterol	Prostaglandin G/H synthase 1
11	MOL000449	Stigmasterol	Prostaglandin G/H synthase 2
12	MOL000449	Stigmasterol	Alpha-2A adrenergic receptor
13	MOL000449	Stigmasterol	Sodium-dependent noradrenaline transporter
14	MOL000449	Stigmasterol	Sodium-dependent dopamine transporter
15	MOL000449	Stigmasterol	Beta-2 adrenergic receptor
16	MOL000449	Stigmasterol	Aldose reductase
17	MOL000449	Stigmasterol	Urokinase-type plasminogen activator
18	MOL000449	Stigmasterol	Leukotriene A-4 hydrolase
19	MOL000449	Stigmasterol	Amine oxidase [flavin-containing] B
20	MOL000449	Stigmasterol	Amine oxidase [flavin-containing] A
21	MOL000449	Stigmasterol	cAMP-dependent protein kinase catalytic subunit alpha

22	MOL000449	Stigmasterol	Chymotrypsinogen B
23	MOL000449	Stigmasterol	Muscarinic acetylcholine receptor M3
24	MOL000449	Stigmasterol	Muscarinic acetylcholine receptor M1
25	MOL000449	Stigmasterol	Beta-1 adrenergic receptor
26	MOL000449	Stigmasterol	Sodium channel protein type 5 subunit alpha
27	MOL000449	Stigmasterol	5-hydroxytryptamine 2A receptor
28	MOL000449	Stigmasterol	Alpha-1A adrenergic receptor
29	MOL000449	Stigmasterol	Gamma-aminobutyric-acid receptor subunit alpha-3
30	MOL000449	Stigmasterol	Muscarinic acetylcholine receptor M2
31	MOL000449	Stigmasterol	Alpha-1B adrenergic receptor
32	MOL000449	Stigmasterol	Gamma-aminobutyric-acid receptor subunit alpha-1
33	MOL000449	Stigmasterol	Neuronal acetylcholine receptor subunit alpha-7
34	MOL004687	2-octanone	Arachidonate 5-lipoxygenase
35	MOL000485	TMH	Gamma-aminobutyric-acid receptor subunit alpha-2
36	MOL000485	TMH	Gamma-aminobutyric-acid receptor subunit alpha-5
37	MOL000485	TMH	Gamma-aminobutyric-acid receptor subunit alpha-1
38	MOL000485	TMH	Cytochrome P450-cam
39	MOL000485	TMH	Gamma-aminobutyric-acid receptor subunit alpha-6
40	MOL000485	TMH	Prostaglandin G/H synthase 2
41	MOL000485	TMH	cAMP-dependent protein kinase catalytic subunit alpha
42	MOL000485	TMH	Neuronal acetylcholine receptor subunit alpha-2
43	MOL005344	ginsenoside rh2	Apoptosis regulator BAX
44	MOL005344	ginsenoside rh2	Tumor necrosis factor
45	MOL005344	ginsenoside rh2	Caspase-3
46	MOL005344	ginsenoside rh2	Prostaglandin G/H synthase 2
47	MOL005344	ginsenoside rh2	NF-kappa-B inhibitor alpha
48	MOL005344	ginsenoside rh2	Interleukin-1 beta
49	MOL005344	ginsenoside rh2	Caspase-1

50	MOL005344	ginsenoside rh2	Interferon gamma
51	MOL005344	ginsenoside rh2	Pituitary adenylate cyclase-activating polypeptide
52	MOL005344	ginsenoside rh2	Proteasome assembly chaperone 1
53	MOL005344	ginsenoside rh2	Dual specificity mitogen-activated protein kinase kinase 4
54	MOL005344	ginsenoside rh2	Solute carrier family 2, facilitated glucose transporter member 4
55	MOL000908	beta-elemene	Prostaglandin G/H synthase 2
56	MOL000908	beta-elemene	Gamma-aminobutyric-acid receptor subunit alpha-2
57	MOL000908	beta-elemene	Retinoic acid receptor RXR-alpha
58	MOL000908	beta-elemene	Sodium-dependent noradrenaline transporter
59	MOL000908	beta-elemene	Gamma-aminobutyric-acid receptor subunit alpha-3
60	MOL000908	beta-elemene	Muscarinic acetylcholine receptor M2
61	MOL000908	beta-elemene	Gamma-aminobutyric-acid receptor subunit alpha-1
62	MOL000908	beta-elemene	Gamma-aminobutyric-acid receptor subunit alpha-6
63	MOL000908	beta-elemene	Prostaglandin G/H synthase 1
64	MOL000908	beta-elemene	Muscarinic acetylcholine receptor M3
65	MOL000908	beta-elemene	Muscarinic acetylcholine receptor M1
66	MOL000908	beta-elemene	Alpha-1A adrenergic receptor
67	MOL000908	beta-elemene	Neuronal acetylcholine receptor subunit alpha-7
68	MOL000908	beta-elemene	Nuclear receptor coactivator 2
69	MOL000908	beta-elemene	Gamma-aminobutyric-acid receptor subunit alpha-5
70	MOL000908	beta-elemene	Apoptosis regulator Bcl-2
71	MOL000908	beta-elemene	Cyclin-dependent kinase inhibitor 1
72	MOL000908	beta-elemene	Eukaryotic translation initiation factor 6
73	MOL000908	beta-elemene	Retinoblastoma-associated protein
74	MOL000908	beta-elemene	Cellular tumor antigen p53
75	MOL000908	beta-elemene	Telomerase protein component 1
76	MOL000908	beta-elemene	Protein CBFA2T1
77	MOL000908	beta-elemene	Cell division control protein 2 homolog

78	MOL000908	beta-elemene	G2/mitotic-specific cyclin-B1
79	MOL000908	beta-elemene	Transforming protein RhoA
80	MOL005843	1-Hydroxycumene	Lysozyme
81	MOL005843	1-Hydroxycumene	Nicotinate-nucleotide--dimethylbenzimidazole phosphoribosyl-transferase
82	MOL000612	(-)-alpha-cedrene	Muscarinic acetylcholine receptor M3
83	MOL000612	(-)-alpha-cedrene	Prostaglandin G/H synthase 2
84	MOL000612	(-)-alpha-cedrene	Gamma-aminobutyric-acid receptor subunit alpha-2
85	MOL000612	(-)-alpha-cedrene	Retinoic acid receptor RXR-alpha
86	MOL000612	(-)-alpha-cedrene	Gamma-aminobutyric-acid receptor subunit alpha-1
87	MOL000612	(-)-alpha-cedrene	Nuclear receptor coactivator 2
88	MOL000612	(-)-alpha-cedrene	Muscarinic acetylcholine receptor M1
89	MOL000066	alloaromadedrene	Muscarinic acetylcholine receptor M3
90	MOL000066	alloaromadedrene	Muscarinic acetylcholine receptor M2
91	MOL000066	alloaromadedrene	Gamma-aminobutyric-acid receptor subunit alpha-1
92	MOL000066	alloaromadedrene	Muscarinic acetylcholine receptor M1
93	MOL000066	alloaromadedrene	Neuronal acetylcholine receptor subunit alpha-2
94	MOL000666	hexanal	Tumor necrosis factor
95	MOL000666	hexanal	Transcription factor AP-1
96	MOL000666	hexanal	Tissue factor
97	MOL000666	hexanal	Mitogen-activated protein kinase 10
98	MOL000666	hexanal	Abl interactor 1
99	MOL000666	hexanal	Platelet glycoprotein 4
100	MOL000675	oleic acid	Prostaglandin G/H synthase 1
101	MOL000675	oleic acid	Nuclear receptor coactivator 2
102	MOL000675	oleic acid	Prostaglandin G/H synthase 2
103	MOL000675	oleic acid	Lysozyme
104	MOL000675	oleic acid	Nicotinate-nucleotide--dimethylbenzimidazole phosphoribosyl-transferase

105	MOL000675	oleic acid	Trypsin-3
106	MOL000675	oleic acid	Retinoic acid receptor RXR-alpha
107	MOL000675	oleic acid	Cytochrome P450-cam
108	MOL000675	oleic acid	Urokinase-type plasminogen activator
109	MOL000675	oleic acid	Superoxide dismutase [Cu-Zn]
110	MOL000675	oleic acid	Telomerase protein component 1
111	MOL000675	oleic acid	Endothelin-1
112	MOL000675	oleic acid	Receptor tyrosine-protein kinase erbB-2
113	MOL000675	oleic acid	Peroxisome proliferator-activated receptor gamma
114	MOL000675	oleic acid	Lipoprotein lipase
115	MOL000675	oleic acid	Neuromodulin
116	MOL000675	oleic acid	Plasminogen activator inhibitor 1
117	MOL000675	oleic acid	Brain-derived neurotrophic factor
118	MOL000675	oleic acid	3-hydroxy-3-methylglutaryl-coenzyme A reductase
119	MOL000675	oleic acid	Myeloperoxidase
120	MOL000675	oleic acid	Peroxisome proliferator-activated receptor alpha
121	MOL000675	oleic acid	Peroxisome proliferator-activated receptor delta
122	MOL000675	oleic acid	C-reactive protein
123	MOL000675	oleic acid	Serum paraoxonase/arylesterase 1
124	MOL000675	oleic acid	Insulin
125	MOL000675	oleic acid	Plasminogen
126	MOL000675	oleic acid	Fatty acid-binding protein, liver
127	MOL000675	oleic acid	Retinol-binding protein 2
128	MOL000675	oleic acid	Glucagon
129	MOL000675	oleic acid	Glutamyl aminopeptidase
130	MOL000675	oleic acid	Mitochondrial uncoupling protein 2
131	MOL000675	oleic acid	Sterol O-acyltransferase 1
132	MOL000675	oleic acid	Cholecystokinin

133	MOL000675	oleic acid	Cbp/p300-interacting transactivator 1
134	MOL000675	oleic acid	Pancreas/duodenum homeobox protein 1
135	MOL000675	oleic acid	Solute carrier family 2, facilitated glucose transporter member 2
136	MOL000675	oleic acid	Peptidyl-glycine alpha-amidating monooxygenase
137	MOL000675	oleic acid	Acyl-CoA desaturase
138	MOL000675	oleic acid	Mitochondrial uncoupling protein 3
139	MOL000675	oleic acid	Cholesteryl ester transfer protein
140	MOL000675	oleic acid	Peptide YY
141	MOL000675	oleic acid	Aspartyl aminopeptidase
142	MOL000675	oleic acid	Cell-death-related nuclease 7
143	MOL000069	palmitic acid	Cathepsin D
144	MOL000069	palmitic acid	Prostaglandin G/H synthase 1
145	MOL000069	palmitic acid	Prostaglandin G/H synthase 2
146	MOL000069	palmitic acid	Rhodopsin
147	MOL000069	palmitic acid	Ig gamma-1 chain C region
148	MOL000069	palmitic acid	Ferrichrome-iron receptor
149	MOL000069	palmitic acid	Nuclear receptor coactivator 2
150	MOL000069	palmitic acid	Apoptosis regulator Bcl-2
151	MOL000069	palmitic acid	Interleukin-10
152	MOL000069	palmitic acid	Tumor necrosis factor
153	MOL000069	palmitic acid	Phosphatidylinositol-3,4,5-trisphosphate 3-phosphatase and dual-specificity protein phosphatase PTEN
154	MOL000069	palmitic acid	Putative beta-glucuronidase-like protein SMA3
155	MOL000069	palmitic acid	Solute carrier family 22 member 5
156	MOL000069	palmitic acid	Choline-phosphate cytidylyltransferase A
157	MOL007459	[(1R)-1-methoxyethyl]benzene	Sodium-dependent noradrenaline transporter
158	MOL007459	[(1R)-1-methoxyethyl]benzene	Alpha-1A adrenergic receptor

159	MOL007459	[(1R)-1-methoxyethyl]benzene	Sodium-dependent dopamine transporter
160	MOL007459	[(1R)-1-methoxyethyl]benzene	Lysozyme
161	MOL007460	Cyclooctadiene	Trypsin-3
162	MOL007461	1,2-DIHYDRO-1,5,8-TRIMETHYLNAPHTHALENE	Nitric oxide synthase, inducible
163	MOL007461	1,2-DIHYDRO-1,5,8-TRIMETHYLNAPHTHALENE	Prostaglandin G/H synthase 1
164	MOL007461	1,2-DIHYDRO-1,5,8-TRIMETHYLNAPHTHALENE	Muscarinic acetylcholine receptor M3
165	MOL007461	1,2-DIHYDRO-1,5,8-TRIMETHYLNAPHTHALENE	Muscarinic acetylcholine receptor M1
166	MOL007461	1,2-DIHYDRO-1,5,8-TRIMETHYLNAPHTHALENE	Prostaglandin G/H synthase 2
167	MOL007461	1,2-DIHYDRO-1,5,8-TRIMETHYLNAPHTHALENE	Nitric-oxide synthase, endothelial
168	MOL007461	1,2-DIHYDRO-1,5,8-TRIMETHYLNAPHTHALENE	Sodium-dependent noradrenaline transporter
169	MOL007461	1,2-DIHYDRO-1,5,8-TRIMETHYLNAPHTHALENE	Alpha-1A adrenergic receptor
170	MOL007461	1,2-DIHYDRO-1,5,8-TRIMETHYLNAPHTHALENE	Beta-2 adrenergic receptor
171	MOL007461	1,2-DIHYDRO-1,5,8-TRIMETHYLNAPHTHALENE	Alpha-1D adrenergic receptor
172	MOL007461	1,2-DIHYDRO-1,5,8-TRIMETHYLNAPHTHALENE	Gamma-aminobutyric-acid receptor subunit alpha-1
173	MOL007461	1,2-DIHYDRO-1,5,8-TRIMETHYLNAPHTHALENE	Amine oxidase [flavin-containing] B

174	MOL007461	1,2-DIHYDRO-1,5,8-TRIMETHYLNAPHTHALENE	cGMP-inhibited 3',5'-cyclic phosphodiesterase A
175	MOL007461	1,2-DIHYDRO-1,5,8-TRIMETHYLNAPHTHALENE	Alpha-1B adrenergic receptor
176	MOL007462	1,4,6-trimethyl-1,2,3,4-tetralin	Prostaglandin G/H synthase 1
177	MOL007462	1,4,6-trimethyl-1,2,3,4-tetralin	Muscarinic acetylcholine receptor M3
178	MOL007462	1,4,6-trimethyl-1,2,3,4-tetralin	Prothrombin
179	MOL007462	1,4,6-trimethyl-1,2,3,4-tetralin	Muscarinic acetylcholine receptor M1
180	MOL007462	1,4,6-trimethyl-1,2,3,4-tetralin	Sodium channel protein type 5 subunit alpha
181	MOL007462	1,4,6-trimethyl-1,2,3,4-tetralin	Prostaglandin G/H synthase 2
182	MOL007462	1,4,6-trimethyl-1,2,3,4-tetralin	Acetylcholinesterase
183	MOL007462	1,4,6-trimethyl-1,2,3,4-tetralin	Sodium-dependent noradrenaline transporter
184	MOL007462	1,4,6-trimethyl-1,2,3,4-tetralin	Alpha-1A adrenergic receptor
185	MOL007462	1,4,6-trimethyl-1,2,3,4-tetralin	Alpha-1B adrenergic receptor
186	MOL007462	1,4,6-trimethyl-1,2,3,4-tetralin	Sodium-dependent dopamine transporter
187	MOL007462	1,4,6-trimethyl-1,2,3,4-tetralin	Beta-2 adrenergic receptor
188	MOL007462	1,4,6-trimethyl-1,2,3,4-tetralin	Alpha-1D adrenergic receptor
189	MOL007462	1,4,6-trimethyl-1,2,3,4-tetralin	Sodium-dependent serotonin transporter
190	MOL007462	1,4,6-trimethyl-1,2,3,4-tetralin	Gamma-aminobutyric-acid receptor subunit alpha-1
191	MOL007462	1,4,6-trimethyl-1,2,3,4-tetralin	Dipeptidyl peptidase 4
192	MOL007462	1,4,6-trimethyl-1,2,3,4-tetralin	Leukotriene A-4 hydrolase
193	MOL007462	1,4,6-trimethyl-1,2,3,4-tetralin	Amine oxidase [flavin-containing] B
194	MOL007463	(1R,2S)-1-ethyl-2-methylcyclopropane	Cytochrome P450-cam

195	MOL007463	(1R,2S)-1-ethyl-2-methylcyclopropane	Bacillolysin
196	MOL007463	(1R,2S)-1-ethyl-2-methylcyclopropane	Trypsin-3
197	MOL007464	1-methyl-5-isopropenyl cyclo-hexene	Prostaglandin G/H synthase 2
198	MOL007464	1-methyl-5-isopropenyl cyclo-hexene	Gamma-aminobutyric-acid receptor subunit alpha-1
199	MOL007464	1-methyl-5-isopropenyl cyclo-hexene	Cytochrome P450-cam
200	MOL007466	DICHLOROANILINE	Lysozyme
201	MOL007466	DICHLOROANILINE	Nicotinate-nucleotide--dimethylbenzimidazole phosphoribosyl-transferase
202	MOL007467	2,6-dimethyl-cyclohexanol	Gamma-aminobutyric-acid receptor subunit alpha-1
203	MOL007467	2,6-dimethyl-cyclohexanol	Cholinesterase
204	MOL007467	2,6-dimethyl-cyclohexanol	Cytochrome P450-cam
205	MOL007472	(9Z,12E)-octadeca-9,12-dienoic acid methyl ester	Prostaglandin G/H synthase 1
206	MOL007472	(9Z,12E)-octadeca-9,12-dienoic acid methyl ester	Prostaglandin G/H synthase 2
207	MOL007486	ZINC01532096	Gamma-aminobutyric-acid receptor subunit alpha-1
208	MOL007492	Butylcyclobutane	Trypsin-3
209	MOL007496	isopulegone	Gamma-aminobutyric-acid receptor subunit alpha-2
210	MOL007497	NaPst	Lysozyme
211	MOL007498	10Z,13Z-nonadecadienoic acid	Prostaglandin G/H synthase 1
212	MOL007498	10Z,13Z-nonadecadienoic acid	Prostaglandin G/H synthase 2
213	MOL007500	panaxatriol	Glucocorticoid receptor
214	MOL007500	panaxatriol	Matrix metalloproteinase-9

215	MOL007501	panaxydol	Eukaryotic translation initiation factor 6
216	MOL007501	panaxydol	Caspase-3
217	MOL007501	panaxydol	Brain-derived neurotrophic factor
218	MOL007502	NSC692928	Prostaglandin G/H synthase 1
219	MOL007504	NSC 308879	Glucocorticoid receptor
220	MOL007505	WLN: QR DG	Nicotinate-nucleotide--dimethylbenzimidazole phosphoribosyl-transferase
221	MOL007508	α-cyperene	Muscarinic acetylcholine receptor M3
222	MOL007508	α-cyperene	Gamma-aminobutyric-acid receptor subunit alpha-1
223	MOL007508	α-cyperene	Nuclear receptor coactivator 2
224	MOL007510	α-copaene	Muscarinic acetylcholine receptor M3
225	MOL007510	α-copaene	Muscarinic acetylcholine receptor M1
226	MOL007510	α-copaene	Prostaglandin G/H synthase 2
227	MOL007510	α-copaene	Gamma-aminobutyric-acid receptor subunit alpha-2
228	MOL007510	α-copaene	Retinoic acid receptor RXR-alpha
229	MOL007510	α-copaene	Sodium-dependent noradrenaline transporter
230	MOL007510	α-copaene	Muscarinic acetylcholine receptor M2
231	MOL007510	α-copaene	Gamma-aminobutyric-acid receptor subunit alpha-1
232	MOL007510	α-copaene	Nuclear receptor coactivator 2
233	MOL007510	α-copaene	Gamma-aminobutyric-acid receptor subunit alpha-6
234	MOL007511	(5S)-5-ethyloxolan-2-one	Cytochrome P450-cam
235	MOL000860	stearic acid	Prostaglandin G/H synthase 1
236	MOL000860	stearic acid	Prostaglandin G/H synthase 2
237	MOL000860	stearic acid	Retinoic acid receptor RXR-alpha
238	MOL000860	stearic acid	Nuclear receptor coactivator 2
239	MOL000860	stearic acid	Ig gamma-1 chain C region
240	MOL000860	stearic acid	Transcription factor Sp1
241	MOL000860	stearic acid	Ectonucleotide pyrophosphatase/phosphodiesterase family member 7

242	MOL000879	methyl palmitate	Prostaglandin G/H synthase 1
243	MOL000879	methyl palmitate	Nuclear receptor coactivator 2
244	MOL000879	methyl palmitate	Ig gamma-1 chain C region
245	MOL000879	methyl palmitate	Transcription factor p65
246	MOL000879	methyl palmitate	Interleukin-10
247	MOL000879	methyl palmitate	Tumor necrosis factor
248	MOL000879	methyl palmitate	Interleukin-6
249	MOL000879	methyl palmitate	Prostaglandin G/H synthase 2
250	MOL000879	methyl palmitate	Prostaglandin E2 receptor, EP3 subtype
251	MOL000905	()-beta-Pinene	Muscarinic acetylcholine receptor M1
252	MOL000905	()-beta-Pinene	Gamma-aminobutyric-acid receptor subunit alpha-2
253	MOL000905	()-beta-Pinene	Muscarinic acetylcholine receptor M2
254	MOL000905	()-beta-Pinene	Gamma-aminobutyric-acid receptor subunit alpha-6
255	MOL000905	()-beta-Pinene	Gamma-aminobutyric-acid receptor subunit alpha-1
256	MOL000905	()-beta-Pinene	Cytochrome P450-cam
257	MOL000905	()-beta-Pinene	Prostaglandin G/H synthase 1
258	MOL000905	()-beta-Pinene	Prostaglandin G/H synthase 2
259	MOL000905	()-beta-Pinene	Retinoic acid receptor RXR-alpha
260	MOL000905	()-beta-Pinene	Retinoic acid receptor RXR-gamma
261	MOL000905	()-beta-Pinene	Amine oxidase [flavin-containing] B
262	MOL000905	()-beta-Pinene	Nuclear receptor coactivator 2
263	MOL000905	()-beta-Pinene	Sodium-dependent noradrenaline transporter
264	MOL003127	Germacrene D	Prostaglandin G/H synthase 1
265	MOL003127	Germacrene D	Prostaglandin G/H synthase 2
266	MOL003127	Germacrene D	Sodium-dependent noradrenaline transporter
267	MOL003127	Germacrene D	Nuclear receptor coactivator 2
268	MOL000935	Hepanal	Muscarinic acetylcholine receptor M3
269	MOL000935	Hepanal	Muscarinic acetylcholine receptor M2

270	MOL000935	Hepanal	Ig gamma-1 chain C region
271	MOL000971	Ethylpalmitate	Nuclear receptor coactivator 2
272	MOL000098	quercetin	Prostaglandin G/H synthase 1
273	MOL000098	quercetin	Androgen receptor
274	MOL000098	quercetin	Peroxisome proliferator-activated receptor gamma
275	MOL000098	quercetin	Prostaglandin G/H synthase 2
276	MOL000098	quercetin	Heat shock protein HSP 90-alpha
277	MOL000098	quercetin	Phosphatidylinositol-4,5-bisphosphate 3-kinase catalytic subunit gamma isoform
278	MOL000098	quercetin	Nuclear receptor coactivator 2
279	MOL000098	quercetin	Dipeptidyl peptidase 4
280	MOL000098	quercetin	Aldose reductase
281	MOL000098	quercetin	Trypsin-1
282	MOL000098	quercetin	DNA topoisomerase 2-alpha
283	MOL000098	quercetin	Prothrombin
284	MOL000098	quercetin	Potassium voltage-gated channel subfamily H member 2
285	MOL000098	quercetin	Sodium channel protein type 5 subunit alpha
286	MOL000098	quercetin	Coagulation factor X
287	MOL000098	quercetin	Beta-2 adrenergic receptor
288	MOL000098	quercetin	Stromelysin-1
289	MOL000098	quercetin	cAMP-dependent protein kinase catalytic subunit alpha
290	MOL000098	quercetin	Coagulation factor VII
291	MOL000098	quercetin	Nitric-oxide synthase, endothelial
292	MOL000098	quercetin	Retinoic acid receptor RXR-alpha
293	MOL000098	quercetin	Acetylcholinesterase
294	MOL000098	quercetin	Gamma-aminobutyric-acid receptor subunit alpha-1
295	MOL000098	quercetin	Amine oxidase [flavin-containing] B
296	MOL000098	quercetin	Transcription factor p65
297	MOL000098	quercetin	Epidermal growth factor receptor

298	MOL000098	quercetin	RAC-alpha serine/threonine-protein kinase
299	MOL000098	quercetin	G1/S-specific cyclin-D1
300	MOL000098	quercetin	Apoptosis regulator Bcl-2
301	MOL000098	quercetin	Bcl-2-like protein 1
302	MOL000098	quercetin	Proto-oncogene c-Fos
303	MOL000098	quercetin	Cyclin-dependent kinase inhibitor 1
304	MOL000098	quercetin	Eukaryotic translation initiation factor 6
305	MOL000098	quercetin	Apoptosis regulator BAX
306	MOL000098	quercetin	Caspase-9
307	MOL000098	quercetin	Urokinase-type plasminogen activator
308	MOL000098	quercetin	72 kDa type IV collagenase
309	MOL000098	quercetin	Matrix metalloproteinase-9
310	MOL000098	quercetin	Mitogen-activated protein kinase 1
311	MOL000098	quercetin	Interleukin-10
312	MOL000098	quercetin	Retinoblastoma-associated protein
313	MOL000098	quercetin	Tumor necrosis factor
314	MOL000098	quercetin	Transcription factor AP-1
315	MOL000098	quercetin	Interleukin-6
316	MOL000098	quercetin	Cyclin-dependent kinase inhibitor 2A, isoforms 1/2/3
317	MOL000098	quercetin	Activator of 90 kDa heat shock protein ATPase homolog 1
318	MOL000098	quercetin	Caspase-3
319	MOL000098	quercetin	Cellular tumor antigen p53
320	MOL000098	quercetin	ETS domain-containing protein Elk-1
321	MOL000098	quercetin	NF-kappa-B inhibitor alpha
322	MOL000098	quercetin	Ornithine decarboxylase
323	MOL000098	quercetin	Xanthine dehydrogenase/oxidase
324	MOL000098	quercetin	Caspase-8
325	MOL000098	quercetin	DNA topoisomerase 1

326	MOL000098	quercetin	RAF proto-oncogene serine/threonine-protein kinase
327	MOL000098	quercetin	Superoxide dismutase [Cu-Zn]
328	MOL000098	quercetin	Protein kinase C alpha type
329	MOL000098	quercetin	Interstitial collagenase
330	MOL000098	quercetin	Hypoxia-inducible factor 1-alpha
331	MOL000098	quercetin	Signal transducer and activator of transcription 1-alpha/beta
332	MOL000098	quercetin	Protein CBFA2T1
333	MOL000098	quercetin	Probable E3 ubiquitin-protein ligase HERC5
334	MOL000098	quercetin	Cell division control protein 2 homolog
335	MOL000098	quercetin	78 kDa glucose-regulated protein
336	MOL000098	quercetin	Receptor tyrosine-protein kinase erbB-2
337	MOL000098	quercetin	Peroxisome proliferator-activated receptor gamma
338	MOL000098	quercetin	Acetyl-CoA carboxylase 1
339	MOL000098	quercetin	Heme oxygenase 1
340	MOL000098	quercetin	Cytochrome P450 3A4
341	MOL000098	quercetin	Caveolin-1
342	MOL000098	quercetin	Myc proto-oncogene protein
343	MOL000098	quercetin	Tissue factor
344	MOL000098	quercetin	Gap junction alpha-1 protein
345	MOL000098	quercetin	Cytochrome P450 1A1
346	MOL000098	quercetin	Intercellular adhesion molecule 1
347	MOL000098	quercetin	Interleukin-1 beta
348	MOL000098	quercetin	Small inducible cytokine A2
349	MOL000098	quercetin	E-selectin
350	MOL000098	quercetin	Vascular cell adhesion protein 1
351	MOL000098	quercetin	Prostaglandin E2 receptor, EP3 subtype
352	MOL000098	quercetin	Interleukin-8
353	MOL000098	quercetin	Protein kinase C beta type

354	MOL000098	quercetin	Baculoviral IAP repeat-containing protein 5
355	MOL000098	quercetin	Dual oxidase 2
356	MOL000098	quercetin	Nitric oxide synthase, endothelial
357	MOL000098	quercetin	Heat shock protein beta-1
358	MOL000098	quercetin	Transforming growth factor beta-1
359	MOL000098	quercetin	Maltase-glucoamylase, intestinal
360	MOL000098	quercetin	Interleukin-2
361	MOL000098	quercetin	Nuclear receptor subfamily 1 group I member 2
362	MOL000098	quercetin	Cytochrome P450 1B1
363	MOL000098	quercetin	G2/mitotic-specific cyclin-B1
364	MOL000098	quercetin	Tissue-type plasminogen activator
365	MOL000098	quercetin	Thrombomodulin
366	MOL000098	quercetin	Plasminogen activator inhibitor 1
367	MOL000098	quercetin	Interferon gamma
368	MOL000098	quercetin	Arachidonate 5-lipoxygenase
369	MOL000098	quercetin	Phosphatidylinositol-3,4,5-trisphosphate 3-phosphatase and dual-specificity protein phosphatase PTEN
370	MOL000098	quercetin	Interleukin-1 alpha
371	MOL000098	quercetin	Myeloperoxidase
372	MOL000098	quercetin	DNA topoisomerase 2-alpha
373	MOL000098	quercetin	Neutrophil cytosol factor 1
374	MOL000098	quercetin	ATP-binding cassette sub-family G member 2
375	MOL000098	quercetin	Hyaluronan synthase 2
376	MOL000098	quercetin	Nuclear factor erythroid 2-related factor 2
377	MOL000098	quercetin	NAD(P)H dehydrogenase [quinone] 1
378	MOL000098	quercetin	Poly [ADP-ribose] polymerase 1
379	MOL000098	quercetin	Aryl hydrocarbon receptor
380	MOL000098	quercetin	26S proteasome non-ATPase regulatory subunit 3
381	MOL000098	quercetin	Solute carrier family 2, facilitated glucose transporter member 4

382	MOL000098	quercetin	Collagen alpha-1(III) chain
383	MOL000098	quercetin	DNA gyrase subunit B
384	MOL000098	quercetin	C-X-C motif chemokine 11
385	MOL000098	quercetin	C-X-C motif chemokine 2
386	MOL000098	quercetin	DDB1- and CUL4-associated factor 5
387	MOL000098	quercetin	Nuclear receptor subfamily 1 group I member 3
388	MOL000098	quercetin	Serine/threonine-protein kinase Chk2
389	MOL000098	quercetin	Insulin receptor
390	MOL000098	quercetin	Claudin-4
391	MOL000098	quercetin	Peroxisome proliferator-activated receptor alpha
392	MOL000098	quercetin	Peroxisome proliferator-activated receptor delta
393	MOL000098	quercetin	Heat shock factor protein 1
394	MOL000098	quercetin	C-reactive protein
395	MOL000098	quercetin	C-X-C motif chemokine 10
396	MOL000098	quercetin	Inhibitor of nuclear factor kappa-B kinase subunit alpha
397	MOL000098	quercetin	Osteopontin
398	MOL000098	quercetin	Runt-related transcription factor 2
399	MOL000098	quercetin	Ras association domain-containing protein 1
400	MOL000098	quercetin	Transcription factor E2F1
401	MOL000098	quercetin	Transcription factor E2F2
402	MOL000098	quercetin	Prostatic acid phosphatase
403	MOL000098	quercetin	Cathepsin D
404	MOL000098	quercetin	Insulin-like growth factor-binding protein 3
405	MOL000098	quercetin	Insulin-like growth factor II
406	MOL000098	quercetin	CD40 ligand
407	MOL000098	quercetin	Interferon regulatory factor 1
408	MOL000098	quercetin	Receptor tyrosine-protein kinase erbB-3
409	MOL000098	quercetin	Serum paraoxonase/arylesterase 1

410	MOL000098	quercetin	Type I iodothyronine deiodinase
411	MOL000098	quercetin	Procollagen C-endopeptidase enhancer 1
412	MOL000098	quercetin	Puromycin-sensitive aminopeptidase
413	MOL000098	quercetin	Hexokinase-2
414	MOL000098	quercetin	Homeobox protein Nkx-3.1
415	MOL000098	quercetin	Ras GTPase-activating protein 1
416	MOL000098	quercetin	Peroxidase C1A
417	MOL000098	quercetin	Glutathione S-transferase Mu 1
418	MOL000098	quercetin	Glutathione S-transferase Mu 2

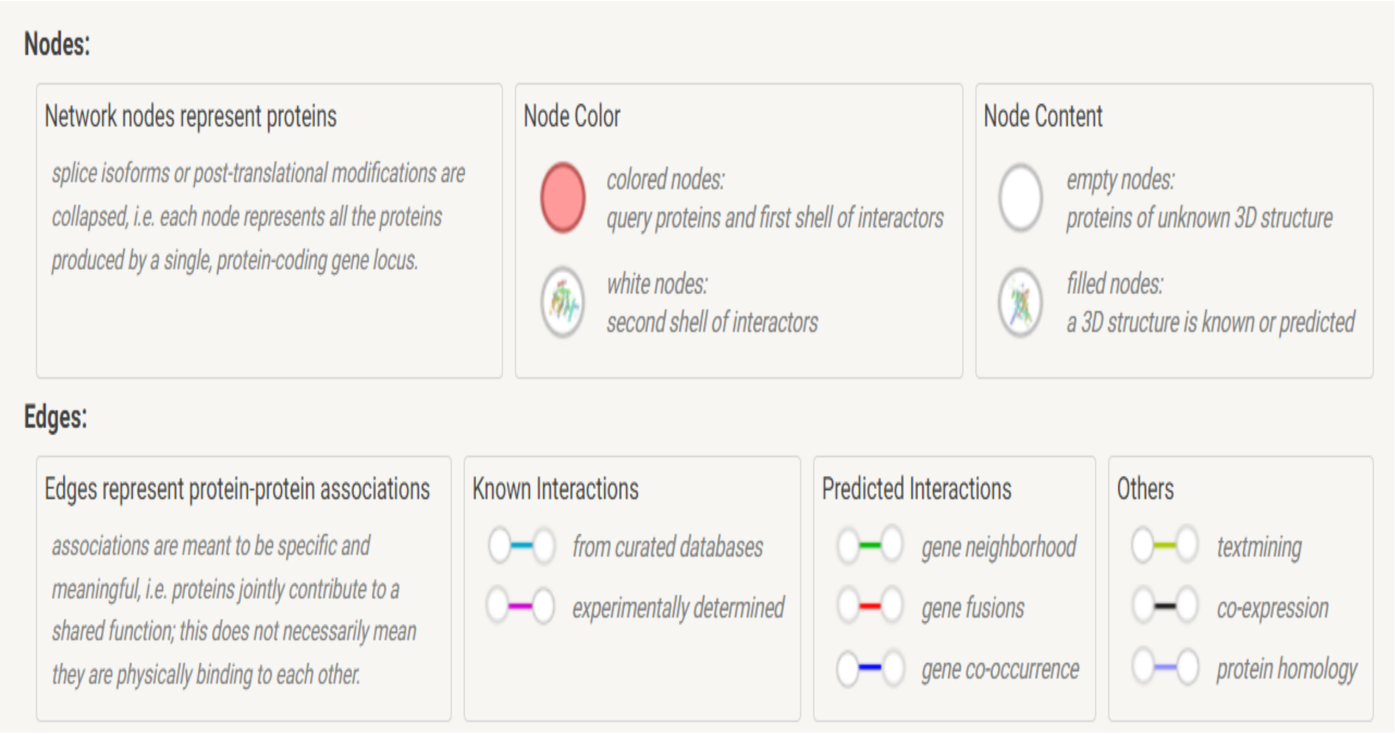

Fig. (3). Additional explanation.
